# Fit in 50 years: participation in high school sports best predicts one’s physical activity after Age 70

**DOI:** 10.1186/1471-2458-13-1100

**Published:** 2013-12-01

**Authors:** Simone Dohle, Brian Wansink

**Affiliations:** 1 Department of Health Science and Technology, Consumer Behavior, ETH Zurich, Universitätsstrasse 22, CHN H75.3, 8092 Zurich, Switzerland; 2 John S. Dyson Professor of Marketing at the Charles H. Dyson School of Applied Economics and Management, Cornell University, 15 Warren Hall, Ithaca NY, USA

**Keywords:** Exercise, Sports, Athletes, Retirement, Veterans, High school athletics, Football, Basketball, Baseball, Track, Elderly, Physical activity

## Abstract

**Background:**

The health benefits of physical activity are widely established, including decreased risk for disease and improved mental well-being. Yet many children, adolescents, and adults do not meet the minimum recommendations specified in current public health guidelines and physical activity is known to decrease during the life course. The aim of this study was to identify background or personality characteristics that predict whether a healthy 25 year-old would become a physically active 75 year-old. This could have powerful implications for targeting physical activity and health interventions.

**Method:**

A unique data set was collected of 712 healthy United States males who passed a rigorous physical exam in the 1940s and who were surveyed 50 years later (in 2000). Their physical activity level after 50 years was correlated and regressed across a wide number of demographic, behavioral, and personality variables from when they were 50 years younger. Data was analyzed in 2012.

**Results:**

In contrast to prior beliefs, self-rated personality profile as a young man had little predictive influence on later-life physical activity. Instead, the single strongest predictor of later-life physical activity was whether he played a varsity sport in high school, and this was also related to fewer self-reported visits to the doctor.

**Conclusion:**

Encouraging systematic or frequent physical activity at a young age - whether through school sports or club opportunities - might be the best investment in long-term activeness. This is relevant at a time when funding for many sports programs is being eliminated and play time is being replaced with screen time.

## Background

The health benefits of physical activity are widely established [1-3]. There is strong evidence that regular, moderate-intensity physical activity reduces the risk of coronary heart disease and stroke, diabetes, hypertension, colon cancer and breast cancer [1-3]. Moreover, research has shown that physical activity not only improves mental well-being, but also is effective treatment for clinical depression and anxiety [4,5].

Despite the well-known positive effects of physical activity on various health outcomes, a broad range of evidence shows that many children, adolescents, and adults do not meet the minimum recommendations specified in current public health guidelines [6-10]. In addition, physical activity is known to decrease during adolescence and adulthood, while sedentary behavior increases [6,11].

To develop effective environmental and policy interventions to increase physical activity in all ages, it is important to understand the correlates and determinants of physical activity and sedentary behavior [9,10,12-14]. For instance, the strongest environmental correlates of physical activity for children (aged 5–13 years depending on the study) have been shown to be walkability, traffıc speed and volume, access and proximity to recreation facilities, residential density and land-use mix (i.e., proximity of homes and destinations such as shops) [12,13]. Residential density and land-use mix were also consistently associated with physical activity in adolescence (aged 12–18 depending on the study) [13]. Regarding psychosocial factors, research has shown that self-efficacy (confidence in the ability to be physically active in specific situations) is a consistent positive determinant of physical activity in children and adolescents [10,12].

Fewer studies investigated how childhood and adolescence activity levels relate to adult activity levels (for a review, see [15]). In a non-concurrent prospective study of men in their mid-twenties, physical fitness test scores measured at both childhood and adolescent age levels were shown to predict physical activity levels as young adults [16]. Related studies point to a similar direction [17,18]. In contrast, however, other studies found that many individuals who were physically active as adolescents became relatively inactive as young adults [6] or showed that levels of physical activity in childhood and 20 years later were only weakly correlated [19,20]. Given these inconclusive findings, researchers have called for the need to identify potential moderators that may influence the tracking of physical activity [6,15]. While one set of these moderators is demographic – ethnicity, gender, or health status – another set of promising predictors might relate to one’s background or personality.

Given the obesity trend, it is important to determine what predicts high – or low – activity levels in adulthood and old age. Not only does physical activity reduce risk of chronic disease and premature death, it also improves emotional, cognitive, social and psychological function in old age [21,22]. Consequently, it is a fundamental question if early physical activity levels influence physical activity in the elderly, also because such relationships are often presumed, but convincing data are lacking.

In a retrospective design, this research examines various long-term determinants of physical activity and health (i.e., self-reported visits to the physician). Taking into account several background, behavioral, and personality variables, the present study focuses on how young men’s physical activity relates to activity and health in old age. In contrast to prior studies, it controls for baseline health status as a potential confounding variable by only including individuals who were markedly healthy as youths.

## Methods

In the 1940s, the United States armed forces decided to screen potential servicemen to determine if they were “fit to fight” in World War II. At that time, malnutrition, physical infirmities or illiteracy precluded 47%^a^ of the drafted or recruited population from serving [23]. Consequently, those who passed the physical exam were considered in top health. By limiting our sample to individuals who fit these two criteria – healthy and male – we hoped to reduce some of the variance that might have been endogenous to prior long-term panel and retrospective studies.

### Sample

This study had approval from the University of Illinois IRB. In 2000, a random national sample of 7,500 World War II veterans was asked to complete a questionnaire were asked to complete some background and personality questions regarding the time before and right after the war, and their current physical activity level. Of the 7,500 questionnaires that were initially mailed, 3,188 were undeliverable (often due to death or incapacitation). One thousand follow-up calls indicated that approximately 53% of the remaining non-respondents were individuals who had passed away, or who could not complete the survey because of health reasons (for details, see [24]). For the present study, only men who were physically healthy at the end of the war were included. Thus, it excludes females and veterans who received a purple heart for military merits, on the assumption that members of the latter group were wounded during the war. In addition, it only included those veterans from World War II who did not serve in any other conflict. The reason for this was that the World War II conscription system could be considered as a universal conscription; deferments were only approved for hardship cases or for those men who worked in the war industry or agriculture. Thus, the social background of men who served in World War II was similar to the non-serving cohort, except that the physical status of the soldiers was better than that of the civilian cohort [23]. This was not the necessarily the case for subsequent conflicts [23]. Deletion of cases which did not meet the inclusion criteria or with missing data on predictor or outcome variables yielded a final sample size of *N* = 712. The mean age was 77.66 (*SD* = 2.34).

### Survey instrument

Each veteran was sent the survey, a cover letter, and a business reply return envelope. The cover letter asked them to complete the survey. For their participation, a small donation was made in their name to the World War II Memorial. They were sent a copy of the major findings of the survey, and they were invited to a symposium that discussed the results.

Respondents were asked a wide range of background questions such as date of birth, how many years they were married to their first spouse, size of the town where they were raised, level of education, credit card use, and whether they were a member of a medical HMO. Some of the answers to the items were non-normal distributed or could not be interpreted directly; thus, they were dummy-coded first. The question about marriage was used to calculate a dummy variable that was given a value of 1 if the respondent was married in 1946 and set to 0 otherwise. The question about size of the town where they were raised was coded into a dummy variable that was given a value of 1 if they responded if the town was larger than 10,000 people. Education was transformed into a dummy variable indicating if the respondent was a college graduate. Credit card use was used as proxy for income; the dummy-coded income variable was set to 1 if a respondent used his credit card more often than the median.

Another group of items related to behavioral variables such as whether they played high school varsity sports, and how many packs of cigarettes they smoked each month in 1946 (after the war was over). The question about smoking was recoded into three categories: non-smokers, light smokers (up to 29 packs each month), and heavy smokers (30 or more packs per month).

To assess the personality variables, they were asked to indicate whether they possessed these traits (1 = strongly disagree; 9 = strongly agree) in 1946 (after the war). The personality traits comprised whether veterans considered themselves to be self-disciplined, to like routine, spontaneous, adventurous, comfortable taking risks, high self-worth, more dominant, restless, and self-centered.

While the use of the International or Global Physical Activity Questionnaire [12,25,26] would have been ideal, there was a concern that respondents would find it too lengthy or irrelevant for their age group. An alternative set of questions was asked and responses were aggregated into a scale. They were asked to indicate how many times on a typical month they (a) walk in the mall, (b) hunt in season, (c) fish in season, (d) golf in season, and (e) play sports; in addition, they were asked to indicate how many hours a week they spend (f) walking for exercise. Because most answers were highly skewed and transformation was unsuccessful, answers were dummy coded (with 0 = “never does the activity” and 1 = “does activity”). The sum of the answers to the six items was aggregated into a scale indicating the individual’s current level of physical activity.

Finally, veterans were asked about their health. Respondents reported how many times they visited their physician in the past year. Because answers to this question were highly skewed, too, it was log-transformed before being analyzed.

## Results

Data was analyzed in 2012. Significant univariate correlates of physical activity and visits to the physician are described in Table 1. Variables that were significantly related to physical activity were varsity sports and non-smoking versus light smoking. Correlations were also noted between physical activity and personality variables (routine, spontaneous, adventurous, comfortable taking risks, dominant, and restless). Significant univariate correlates of visits to the physician were income, varsity sports, and non-smoking versus heavy smoking, but no univariate correlation was found between personality variables and visits to the physician.

**Table 1:** Correlates of future physical activity and physician visits

	**Physical activity**	**Visits to the physician**
Age	.024	.066
Married	.004	-.001
Size town raised	.029	.050
College	-.024	.013
Income	.023	.128^**^
HMO member	-.047	.014
Varsity sports	.164^**^	-.085^*^
Non-smoking vs. light smoking	.082^*^	-.053
Non-smoking vs. heavy smoking	-.036	.115^**^
Self-disciplined	-.071	.026
Routine	-.091^*^	.051
Spontaneous	.077^*^	.016
Adventurous	.169^**^	.067
Comfortable taking risks	.081^*^	.016
High self-worth	-.018	.004
More dominant	.091^*^	.052
Restless	.095^*^	.019
Self-centered	.068	.017

*p < .05 **p < .01. The Spearman rank correlation coefficient was used.

Data collected in 2000 in the United States.

Multiple linear regression analysis was used to determine the independent effects of the predictor variables on physical activity (see Table 2). Two variables showed a significant effect. Varsity sport was the strongest predictor (*β* = 0.173, *p* < .01). Men who played varsity sport in high school were more likely to be physically active in old age. In addition, respondents who were more adventurous were also more physically active (*β* = 0.164, *p* < .01).

**Table 2:** Multiple linear regression predicting physical activity

	***B***	***SE B***	**β**	***P*-*value***
Constant	-.563	1.639		.732
Background				
Age	.027	.021	.050	.188
Married	.023	.103	.008	.823
Size of town raised	.143	.096	.056	.138
College education	-.100	.100	-.038	.319
Income	.018	.095	.007	.846
HMO member	-.157	.099	-.058	.113
Behavioral				
Varsity sport	.447	.096	.173	.001**
Smoking				
Non-smoking vs. light smoking	.186	.122	.062	.127
Non-smoking vs. heavy smoking	-.058	.109	-.022	.594
Personality				
Self-disciplined	-.027	.032	-.038	.400
Routine	-.043	.024	-.073	.070
Spontaneous	.003	.029	.005	.912
Adventurous	.100	.029	.164	.001**
Comfortable taking risks	.002	.028	.003	.945
High self-worth	-.027	.032	-.036	.403
More dominant	.027	.028	.042	.340
Restless	.020	.023	.035	.387
Self-centered	.008	.025	.013	.751

**p < .01.

Adjusted *R*^*2*^ = .06.

Data collected in 2000 in the United States.

The relative importance of the predictor variables to visits to the physician (log-transformed) was determined by a second multiple linear regression analysis. Table 3 gives an overview of the results of the analysis. In the multivariate regression, the largest predictor of visits to the physician was income: respondents with higher income went to the physician more often (*β* = 0.143, *p* < .01). Another significant predictor was smoking. Men who were heavy smokers in 1946 visited the doctor more often fifty years later than men who were non-smokers in 1946 (*β* = 0.114, *p* < .01). Varsity sport was inversely related to visits to the physician; thus, men who played varsity sports visited the doctor less often (*β* = -0.102, *p* < .01). A positive association was also found between age and visits to the physician (*β* = 0.100, *p* = .01). Finally, being adventurous was also a significant predictor, and positively related to visits to the physician (*β* = 0.119, *p* < .05).

**Table 3:** Multiple linear regression predicting visits to the physician

	***B***	***SE B***	**β**	***P*-*value***
Constant	-.437	.433		.313
Background				
Age	.014	.005	.100	.010*
Married	-.006	.027	-.009	.813
Size of town raised	.014	.025	.021	.571
College education	.009	.026	.013	.728
Income	.096	.025	.143	.001**
HMO member	.018	.026	.025	.495
Behavioral				
Varsity sport	-.069	.025	-.102	.007**
Smoking				
Non-smoking vs. light smoking	-.018	.032	-.023	.569
Non-smoking vs. heavy smoking	.079	.029	.114	.006**
Personality				
Self-disciplined	-.002	.008	-.010	.834
Routine	.009	.006	.058	.155
Spontaneous	-.009	.008	-.051	.247
Adventurous	.019	.008	.119	.014*
Comfortable taking risks	-.004	.007	-.022	.616
High self-worth	-.007	.009	-.034	.437
More dominant	.009	.007	.052	.240
Restless	.001	.006	.007	.859
Self-centered	-.001	.007	-.008	.841

*p < .05 **p < .01.

Adjusted *R*^*2*^ = .04.

Data collected in 2000 in the United States.

## Discussion

It is often presumed that physical activity and personality in childhood and adolescence predicts physical activity in adulthood, or even old age. This research sought to more exactingly test the presumption that physical activity at a young age predicts physical activity at an elderly age. Importantly, this sample consisted of World War II veterans; therefore, it only included men who were in good health in their youth. This sample was representative of the entire age cohort, since the United States conscription system was fairly universal and inducted “…the chemist and the street cleaner, the physicist and the plumber, the biologist and the bookie” (Flynn, 1993, page 105).

These results revealed one critical youthful predictor of whether a man would be physically active after the age of 70: It was whether he participated in high school varsity sports. To our knowledge, no study has documented an influence of varsity sport on later physical activity over such a long time period, especially in a population that had already been prescreened as healthy, physically fit at a young age.

Despite the fact that recent studies found that environmental attributes are related to activity levels [12,13] this study found no effect of the environment; the size of the town where a person was raised was not related to physical activity. Town size affects environmental attributes such as traffıc speed and volume, or access and proximity to recreation facilities; it should be noted, however, that these attributes were not measured directly in the present study. Likewise, personality variables seem to have little bearing on physical activity in old age. Self-reported personality traits such as being adventurous, comfortable taking risks or being dominant showed some relation to physical activity in old age when considered in univariate analyses, but only being adventurous contributed a significant independent effect in the multivariate analysis.

The current study also shows that high school varsity sport is not only related to activity levels in old age, but also to health status in old age. Smoking as another behavioral variable also showed a strong influence on health. Individuals who were heavy smokers immediately after the war reported more frequent present-day doctor visits than non-smokers, pointing to the importance of public health interventions aimed to prevent smoking.

Other predictors of long-term health were age, income, and again, being adventurous. It should be noted that being adventurous was positively related to visits to the doctor in old age, suggesting that being adventurous may foster physical activity, but probably also other, risky behaviors. Indeed, there is evidence that adventure-seeking as part of a risk-taking personality is related to risky behavior, specifically to alcohol consumption and delinquency, and to a lesser extent, to drinking and driving, risky driving, and drug use [27]. Health practitioners or educators are therefore advised not to encourage overly adventurous behavior in adolescents.

### Limitations and future research

Investigating the 50-year impact of different background, behavioral, and personality variables has methodological limitations. First, the design used retrospective questioning, which may involve selective recall or problems to retrieve information from memory [28,29]. In addition, while much research suggests that self-reported characteristics are quite stable throughout life [30], there may be biases in some of these measures [31]. For example, self-perception theory [32] states that people assume they possess certain characteristics by observing their own behavior. It could be that the men in this study simply inferred that they had certain characteristics. Further longitudinal research is warranted, using more extensive measures of personality as well as early life physical activity that allow differentiation between competitive sport and recreational physical activity.

It is also important to acknowledge that the present study is limited to healthy young men, and confounding by variables not controlled for cannot be completely excluded. A direction for further research is to delve deeper into the generalizability of the present results to other samples (e.g. females, less healthy populations).

## Conclusions

These findings offer some compelling suggestions about how to target young adults at risk for long-term adult inactivity, chronic diseases, and premature death. In particular, these are youth who are not involved in organized sports (such as varsity athletics).

The first suggestion would be to maintain or enhance high school athletic programs, even in an era of shrinking school budgets. It has been noted that physical education classes may be the only opportunity for many adolescents to engage in weekly physical activity [33]. School-based organized sports should be preserved because they contribute to later physical activity levels and decrease the risk factors for early morbidity. Perhaps there are more cost-effective ways to maintain sports programs without eliminating them.

Second, it might be that other forms of relatively vigorous exercise and physical education classes could be promoted across grade levels. They need not concentrate on competition but rather on enjoyment, and on the benefits of and ways to stay physically active over the lifespan [34,35]. Finally, parents and other caregivers play a role in encouraging children to be active [9,10,36,37] – including sports leagues and other exercises activities at youth centers, free or low-cost municipal summer camps, or YMCA’s [34]. Less competitive children can be steered away from the more competitive sports and guided towards non-competitive forms of running, dance, swimming, weight lifting, or martial arts [34].

## Endnote

^a^During World War II, the United States Selective Service System called up 19 million men. Nine million men were rejected because they didn’t pass the double examination by local physicians and army induction centers [23].

## Competing interests

The authors declare that they have no competing interests.

## Authors’ contributions

BW conceived of and conducted the study. SD analyzed and interpreted the data and drafted the manuscript. BW assisted in the interpretation of the data and provided critical feedback on drafts. SD and BW read and approved the final manuscript.

## Pre-publication history

The pre-publication history for this paper can be accessed here:

http://www.biomedcentral.com/1471-2458/13/1100/prepub
